# Nerve Fiber Bundle Damage in Spinocerebellar Degeneration on Diffusion Tensor Imaging

**DOI:** 10.2174/0115734056385511250912062114

**Published:** 2025-09-23

**Authors:** Hong-Xin Jiang, Yan-Mei Ju, Guo-Min Ji, Ting-Ting Gao, Yan Xu, Shu-Man Han, Lei Cao, Jin-Xu Wen, Hui-Zhao Wu, Bulang Gao, Wen-Juan Wu

**Affiliations:** 1 Hebei Provincial Gucheng County People’s Hospital, Gucheng, China; 2 Department of Radiology, Hebei Medical University Third Hospital, Shijiazhuang, China

**Keywords:** Diffusion tensor imaging, Spinocerebellar degeneration, Cerebellar arcuate fibers, Corticocerebellar tract, Middle cerebellar peduncle, MRI

## Abstract

**Introduction:**

This study aimed to investigate nerve fiber bundle damage associated with spinocerebellar degeneration, a dominant inherited neurological disorder, using magnetic resonance imaging (MRI) with diffusion tensor imaging (DTI).

**Methods:**

Four cases of spinocerebellar degeneration and ten matched healthy subjects were retrospectively enrolled. DTI software was used for processing and analysis.

**Results:**

All patients had an abnormal spinocerebellar ataxia (SCA) type 3 gene mutation, with cerebellar and brainstem atrophy, a decreased signal in the pons and projection fibers. Significant interruption and destruction were revealed in the midline of the cerebellar peduncle, cerebellar arcuate fibers, and the spinothalamic and spinocerebellar tracts. Significant (*p* <0.05) decreases were detected in FA values in the cerebellar peduncle (0.51±0.04 *vs.* 0.68±0.02), cerebellar arcuate fibers (0.37±0.08 *vs.* 0.51±0.05), spinothalamic tract (0.42±0.03 *vs.* 0.49±0.05), and spinocerebellar tract (0.44±0.06 *vs.* 0.52±0.06) compared with healthy controls. Compared with healthy controls, significant (*p* <0.05) increases were detected in ADC values in the cerebellar peduncle (0.84±0.11 *vs.* 0.67±0.03), cerebellar arcuate fibers (0.87±0.12 *vs.* 0.66±0.05), spinothalamic tract (0.89±0.13 *vs.* 0.70±0.03) within the brainstem, and spinocerebellar tract (0.79±0.07 *vs.* 0.69±0.06).

**Discussion:**

The MRI DTI technique provides sufficient information for studying spinocerebellar degeneration and for conducting further research on its etiology and diagnosis. Some limitations were present, including the retrospective and single-center study design, a limited patient sample, and enrollment of only Chinese patients.

**Conclusion:**

The MRI DTI technique can clearly demonstrate the degree of damage to nerve fiber bundles in the cerebellum and the adjacent relationship between the fiber bundles entering and exiting the cerebellum in patients with spinocerebellar degeneration.

## 1 INTRODUCTION

Hereditary ataxias are a group of rare neurodegenerative diseases that affect the cerebellum, its fiber connections, and other fiber bundles of the central nervous system, including the basal ganglia, brainstem, and spinal cord. They can be sub-divided by their mode of inheritance, with the most common being the autosomal dominant spinocerebellar ataxias (SCAs) [1]. Spinocerebellar ataxias are a group of autosomal dominant neurodegenerative diseases that are genetically and clinically heterogeneous, encompassing olivopontocerebellar degene-ration, olivopontocerebellar ataxia, and Friedreich ataxia. This group of diseases is characterized by a range of neurological symptoms, including loss of balance and motor coordination, dysarthria, pyramidal and extrapyramidal signs, oculomotor abnormalities, and other non-ataxia symptoms caused by progressive dysfunction of the cerebellum and its afferent and efferent connections [1-3]. The SCAs include over 40 distinct subtypes, with the most frequent subtypes (SCA1, SCA2, SCA3, SCA6, SCA7, and SCA17) caused by CAG repeat expansion mutations, with the main neuropathological hall-mark being degeneration of Purkinje neurons, consecutive cerebellar atrophy, and involvement of different nervous system regions among particular subtypes [1]. Young patients often have a family history, and the etiology of late-onset sporadic patients is not yet clear. Significant cerebellar atrophy is observed in pathology, with almost all Purkinje cells disappearing under the microscope. Cerebellar atrophy in these patients is mainly limited to the cerebellum and the olivary nucleus. The anatomical structure and function of the cerebellum are complex. The cerebellum is linked to motor coordination and balance, and is involved in higher cognitive functions, including cognition, emotion, and personality [1, 4]. The routing and distribution of nerve fiber bundles in and around the cerebellum are closely related to behavior and function, and have long been a core issue of concern in neuroscience, as well as the basis for localization and qualitative analysis in clinical neurology.

Diffusion tensor imaging (DTI) is the only non-invasive magnetic resonance imaging (MRI) technique that can construct *in vivo* white matter fiber bundles in the brain and track changes in the course of fiber bundles, which reflects the diffusion of water molecules and provides a quantitative description of organization by fractional anisotropy in a three-dimensional and intuitive manner [5-7]. DTI has broad prospects for clinical application. Fractional anisotropy (FA) and apparent diffusion coefficient (ADC, also known as mean diffusivity), derived from DTI, have gained widespread acceptance as sensitive metrics for quantifying microstructural damage to the gray and white matter in neurodegenerative diseases, particularly SCAs, in recent years. The ADC primarily reflects the diffusion velocity of water molecules without showing the movement direction. In nerve degene-ration, nerve cell loss and myelin degeneration increase the movement of water molecules, and the rate of diffusion of water molecules responds accordingly, with anisotropy being decreased. The FA value can indicate the consistency of nerve fibers, structural tightness, and integrity of the microstructure. The evaluation of deep cerebellar nuclei using the DTI technique can serve as a simple imaging indicator of cerebellar disease. This study employed the DTI technique to investigate nerve fiber bundle damage in spinocerebellar degeneration, providing a comprehensive analysis of the fiber bundles that enter and exit the cerebellum. It is of great significance for the precise localization of brain function, especially in clinical diagnosis, treatment planning, and prognosis assessment, with broad application prospects.

## 2 MATERIALS AND METHODS

This retrospective single-center study was approved by the ethics committee of Hebei Provincial Gucheng County Hospital (20220188), and written informed consent was obtained from all participants. The Helsinki Declaration has been followed in the involvement of human subjects in the study, and all human rights have been observed. Patients with spinocerebellar degeneration were enrolled between March 2017 and July 2021. The inclusion criteria were adult patients with spinocerebellar degeneration characterized by an abnormal SCA3 gene, unstable walking, unclear articulation, involuntary hand tremors, choking and coughing during meals, or intermittent dizziness. Patients without spinocerebellar degeneration were excluded. Healthy subjects without any diseases and with matched age were enrolled as the healthy controls for comparison. The data collected were de-identified/anonymized. All patients and healthy controls were from Hebei Provincial Gucheng County Hospital.

### 2.1 Participants

Four patients with spinocerebellar degeneration were enrolled, with the first three patients belonging to the same family. There were two males (50%) and two females (50%) aged 45–65 years (mean 58.7±2). Case 1 was a 55-year-old man who had experienced unstable walking in both lower limbs for 8 years and aggravated dysphagia for 2 years, and the current symptoms included unclear articulation, choking and coughing during meals, stiffness in both lower limbs, inability to stand and walk, involuntary hand tremors, and poor sleep. Case 2 was a 62-year-old woman who had experienced unstable walking for 10 years, and the condition had worsened over the past 10 days. Case 3 was a 65-year-old woman who was unable to walk and speak and had trembling hands. Case 4 was a 65-year-old woman who was admitted because of intermittent dizziness for 2 months, overall stiffness, cogwheel rigidity, and orthostatic hypotension. Ten healthy subjects, matched for age and sex, were enrolled as the control group (mean age, 58.3±7 years), comprising five males (50%) and five females (50%).

### 2.2 Equipment and Methods

The Philips 1.5T conventional MRI scanner was used for DTI scanning, and the subject's head was fixed with a sponge pad before scanning to prevent head movement. The subject was required to remain conscious and had cotton balls placed in both external ear canals. The following coils were selected: a 16-channel neurovascular coil (Sense NV 16 Achieva), an HST Head Coil (Sense HST Multiva), and a DStream Head Coil (dS Head, Ingenia). In the DTI scan, the spin echo planar imaging (SE-EPI) sequence was used with 32 diffusion-sensitive gradient directions, a bipolar diffusion method, 60 layers, a layer thickness of 2.0 mm, interlayer spacing of 0, double b-values, a high b-value of 1000, TR = 10000–12000, TE = 60–85, and an EPI factor of 60. The scanning parameters were: TR = 4800 ms, TE = 85 ms, matrix 128 × 128, NEX = 2, FOV 250 mm × 250 mm, layer thickness = 1.1 mm, without gaps, and b = 0 and 1000 s/mm^2^. The MRI structural scanning parameters were as follows: 176 slices, TR = 1900 ms, TE = 3.44 ms, slice thickness = 1 mm, inversion time (TI) = 900 ms, flip angle = 9°, FOV = 256 mm × 256 mm, and acquisition matrix = 256 × 256. The structural imaging (sMRI) scanning parameters were: sagittal scan, 128 layers, TE = 3.39 ms, TR = 2530 ms, layer thickness = 1.33 mm, interval = 0 mm, TI = 1100 ms, FOV = 256 mm × 256 mm, in-plane resolution = 256 × 192, and FA = 7°. The T1 sequence was used in the sagittal plane scanning, and in the coronal and cross-sectional images, the positioning line was located at the midline plane that coincided with the cerebral falx; the position and angle in the sagittal plane scanning were adjusted using the T1 sequence to center the image.

### 2.3 DTI Image Processing

The scanned DTI raw data were imported into the DTI Studio software on the Extended MR Workstation for smoothing and denoising before being processed to generate images of FA and ADC. The selection of phase encoding direction affects the spatial resolution and artifact distribution of the image, and forward and backward encoding reduces the impact of motion artifacts (such as breathing artifacts) on the image. Thus, the phase encoding direction was selected as forward and backward. When the number of signal averages (NSA) was increased, the signal-to-noise ratio also increased. By averaging multiple signals, random noise was suppressed, but the scanning time increased linearly. Increasing the diffusion gradient direction also made the fiber bundle boundary clearer. In DTI, increasing the number of gradient directions (such as from 6 to 32 directions) can more accurately track the fiber bundle direction, reduce anisotropic artifacts, and make the white matter fiber bundle boundary clearer, but it can also prolong the scanning time. Therefore, in this study, the phase encoding direction was selected as forward and backward, and the NSA and the diffusion gradient direction were increased to make the fiber bundle boundary clearer. The fiber assignment by continuous tracking (FACT) algorithm was applied for whole-brain fiber bundle tracking, with a threshold setting of FA < 0.25. Conduction bundle reconstruction was performed by fusing the obtained DTI image with the conventional 3D FLAIR sequence. The original dataset of diffusion tensor volumes was corrected firstly for eddy current distortions with the FSL FDT diffusion toolbox (FDT; Oxford Centre for Functional Magnetic Resonance Imaging of the Brain (FMRIB)’s Software Library, UK; http://www.fmrib.ox. ac.uk/fsl/fdt/fdt_dtifit.html [8]), and then, the motion artifacts were corrected with the FSL FLIRT (FMRIB’s Linear Image Registration Tool). The diffusion image in each direction was aligned to the b0 volume as the reference image using a 3D rigid body registration algorithm with 6 degrees of freedom and a correlation ratio as the cost function to reduce motion artifacts. The eigenvectors (v1, v2, v3) and eigenvalues (λ1, λ2, λ3) of the diffusion tensor matrix were calculated from the pre-processed DTI volumes for each subject with the FSL FDT diffusion toolbox.

### 2.4 Statistical Analysis

The statistical analysis was performed with the SPSS software (IBM, Chicago, IL, USA). Continuous measurement data were presented as mean and standard deviation, if in a normal distribution, and tested using the t-test. Categorical data were presented in frequency and percentage and tested using the Chi-square test. A significant *p*-value was set at *p* < 0.05.

## 3 RESULTS

All patients had an abnormal SCA3 gene and were mainly characterized by unstable walking. The molecular genetic testing revealed a repeated CAGA count of the SCA3-related gene, with one instance repeated 14 times, which falls within the normal range, and the other repeated 75 times, encom-passing the entire mutation range and consistent with the genetic mutation characteristics of SCA3. The CAG repeat numbers of other subtypes of SCA1, 2, 6, 7, and 12, as well as DRPLA-related genes, were within the normal range. Specific results were: SCA1 (28), SCA2 (22), SCA6 (13; 14), SCA7 (10; 12), SCA12 (16; 18), and DRPLA (10:15). Only one allele was detected in SCA1 and SCA2, which the TP-PCR technology had validated with no abnormal alleles found.

**Fig. (1):**
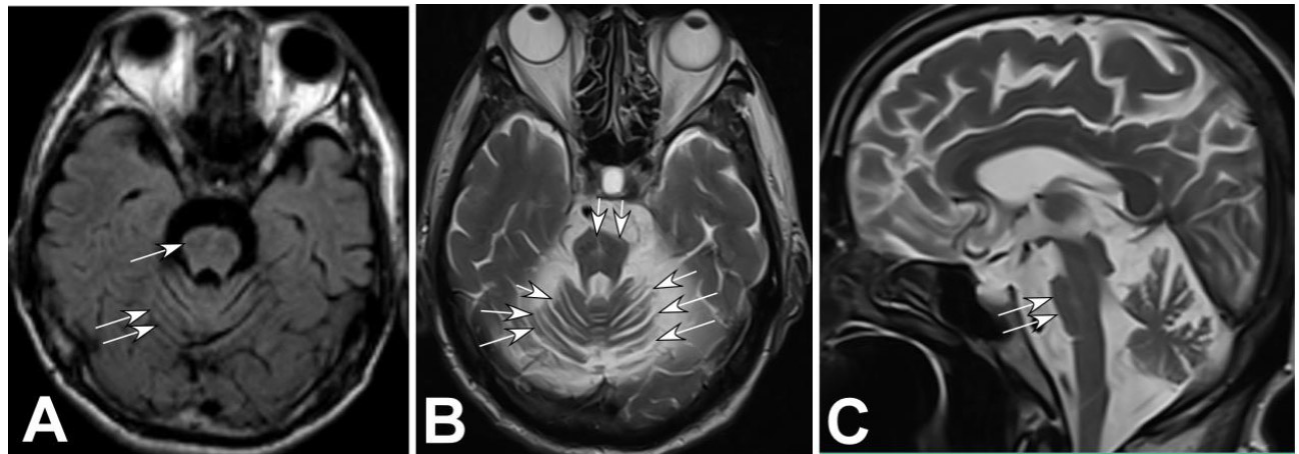
Magnetic resonance imaging (MRI) of the brain in case 1. (**A)**. FLAIR sequence (**A**) showed cerebellar atrophy (double arrows) and thinning of the brainstem (arrow). (**B)**. The top view demonstrates cerebellar atrophy (arrows) and thinning of the brainstem (double arrows). (**C**). Lateral view demonstrated decreased signal at the anterior edge (double arrows) of the pons.

**Fig. (2):**
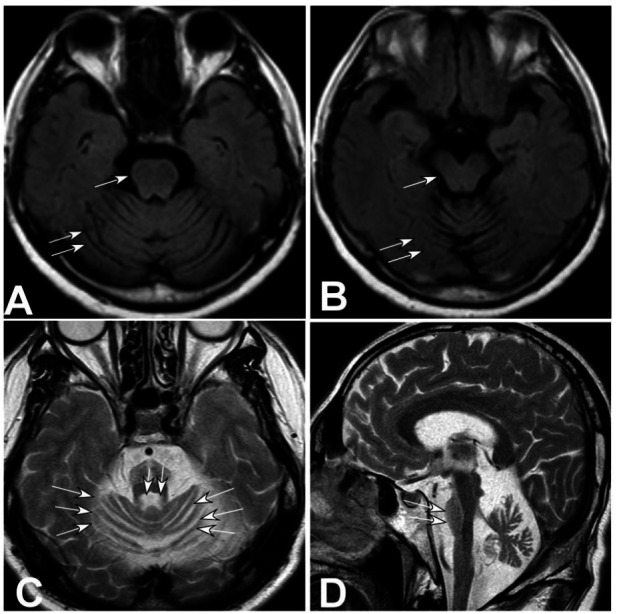
Magnetic resonance imaging (MRI) of the brain in case 2 showing atrophy of the cerebellum, brainstem, pons, and projective fibers. (**A** and **B**). Top view: cerebellar atrophy (double arrows) and thinning of the brainstem (arrow). (**C**). Top view: decreased signal (atrophy) at the anterior edge of the pons (double arrows) and the projection fibers (arrows). (**D**). Lateral view demonstrated atrophy of the pons (double arrows).

**Fig. (3):**
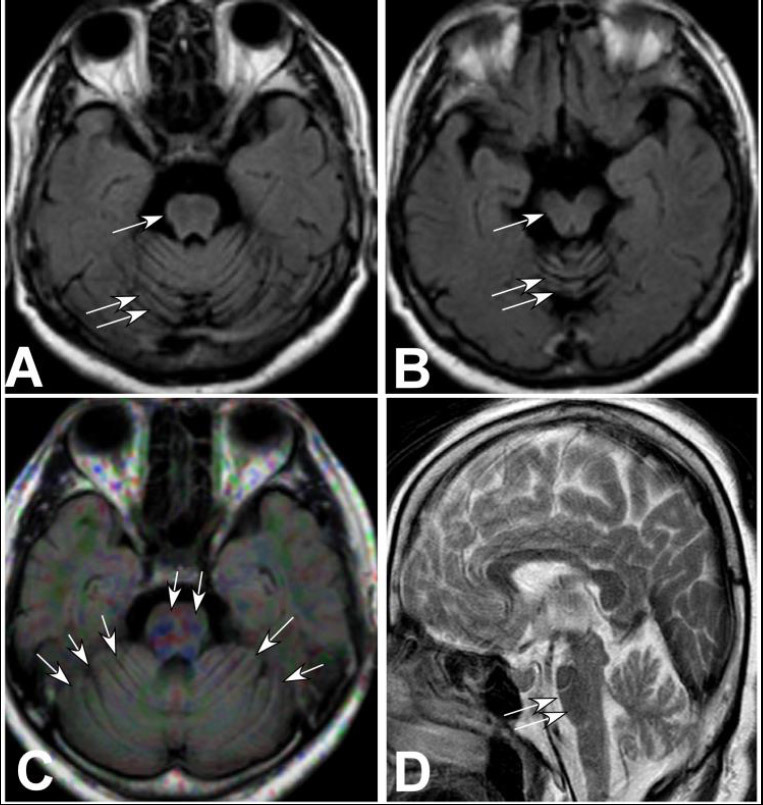
Cerebellar atrophy, brainstem thinning, decreased signal of the pons, and projective fibers in case 3. (**A**) and (**B**). Top view: Cerebellar atrophy (double arrows) and thinning of the brainstem (arrow) were shown. (**C**). The top view showed a decreased signal at the anterior edge of the pons (double arrows) and the projection fibers (arrows). (**D**). Lateral view revealed that the brainstem thinning was mainly in the pons (double arrows).

Brain MRI (Figs. **1**-**3**) showed cerebellar atrophy and thinning of the brainstem (Figs. **1A** and **B**, **2A** and **B**, and **3A** and **B**) [9-12], a decreased signal at the anterior edge of the pons and pale projection fibers (Figs. **1C**, **2C**, and **3C**), and brainstem thinning primarily in the pons (Figs. **2D** and **3D**).

**Fig. (4):**
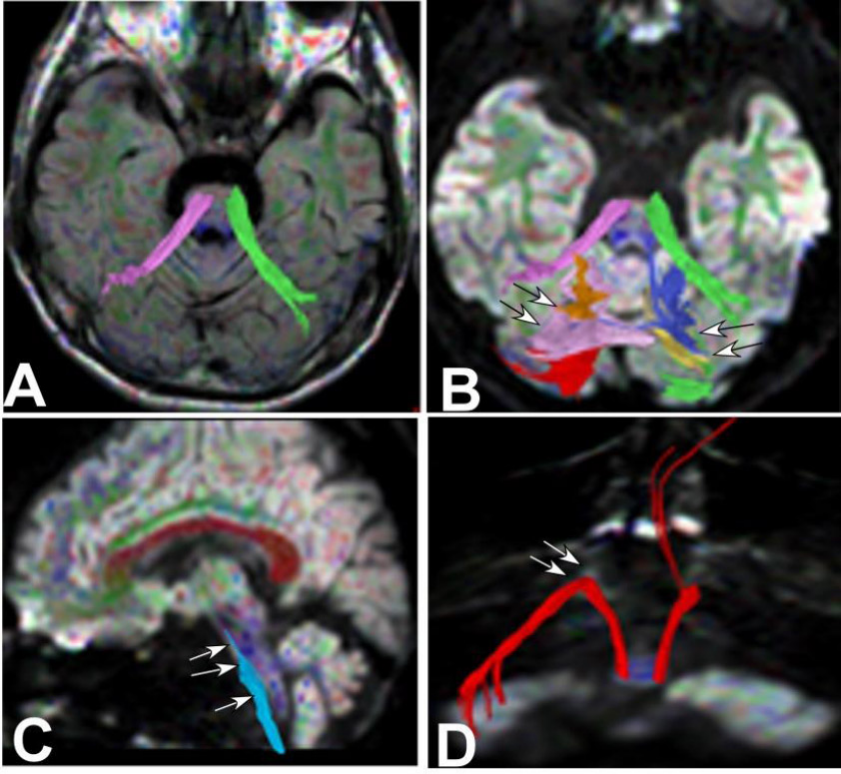
Degeneration of the pons and cerebellum in Case 2. (**A**). The fiber bundles in the midline of the cerebellar peduncle (purple on the right and green on the left) were broken in the top view. (**B**). Bottom view revealed significant interruption and destruction of arcuate fibers in the cerebellum (arrows). (**C**). Left side view demonstrated interruption and destruction of the spinothalamic tract (arrows) within the brainstem because of degeneration of the pons. (**D**). Back view showed that the right spinocerebellar tract (red) was interrupted at the entry site (arrows) into the cerebellum.

**Fig. (5):**
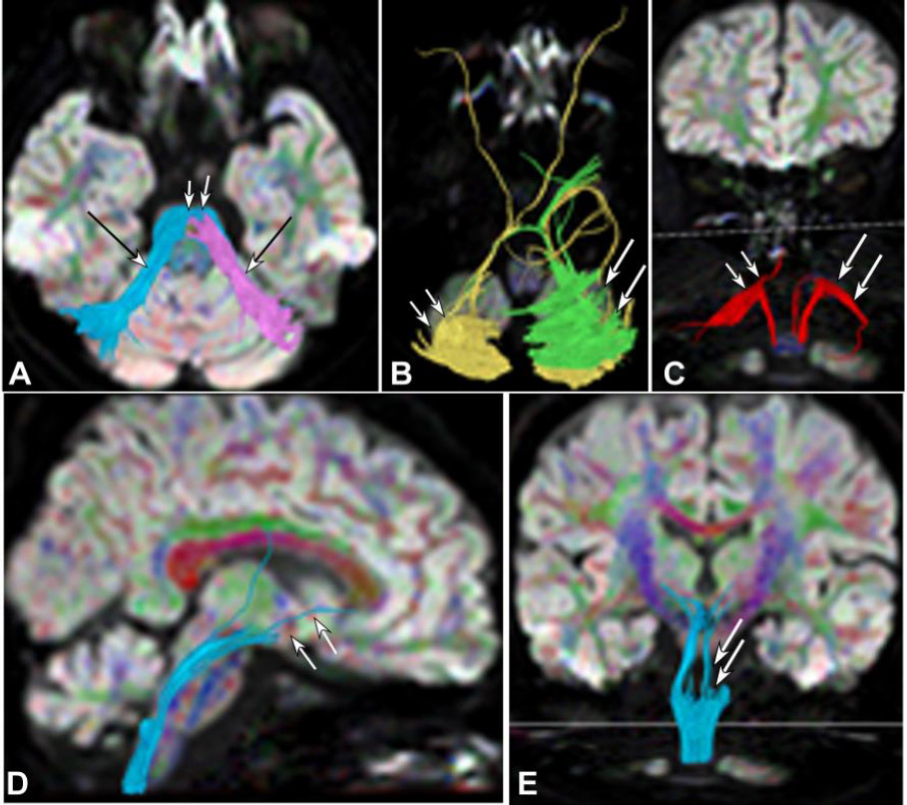
Damages of the cerebellar peduncle, cerebellar hemispheres, spinocerebellar tracts, and spinothalamic tract in case 2. (**A**). Bottom view: An interruption was revealed in the midline of the cerebellar peduncle (blue on the right and purple on the left) on both sides (normal cerebellar peduncle partially connected both cerebellar hemispheres, with some reaching the same cerebellar hemisphere from the midline (double arrows). (**B**) Top view: Significant interruption and destruction of arcuate fibers (yellow and green) in both cerebellar hemispheres were shown. (**C**). Back view: The spinocerebellar tracts (red, double arrows) on both sides experienced relatively mild damage. (**D** and **E**). Right side view (D, blue, double arrows) and back view (E, blue, double arrows): The lesion caused significant disruption and interruption of the spinothalamic tract (blue).

**Fig. (6):**
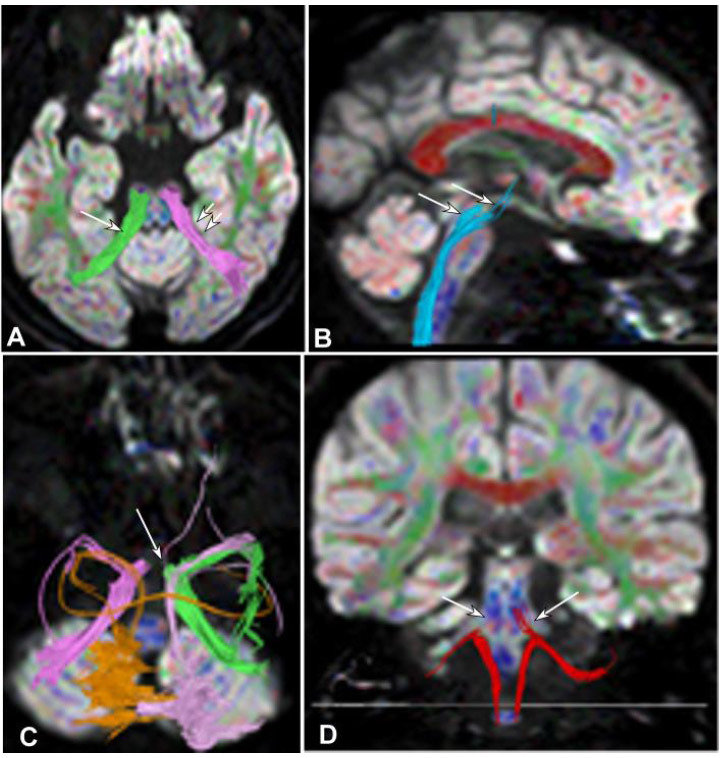
The cerebellar peduncle destruction, spinothalamic tract involvement, and mild damage of the spinocerebellar tract in case 3. (**A**). Top view: Significant interruption and destruction were demonstrated in the midline of the cerebellar peduncle (long arrow on the right and double arrows on the left) on both sides. (**B**). Right side view: The spinothalamic tract (arrows) was damaged. (**C**). Top view: Significant interruption and destruction were shown in the midline (arrow) of the cerebellar peduncle. (**D**). Back view: The spinocerebellar tracts (red) on both sides experienced relatively mild damage.

**Fig. (7):**
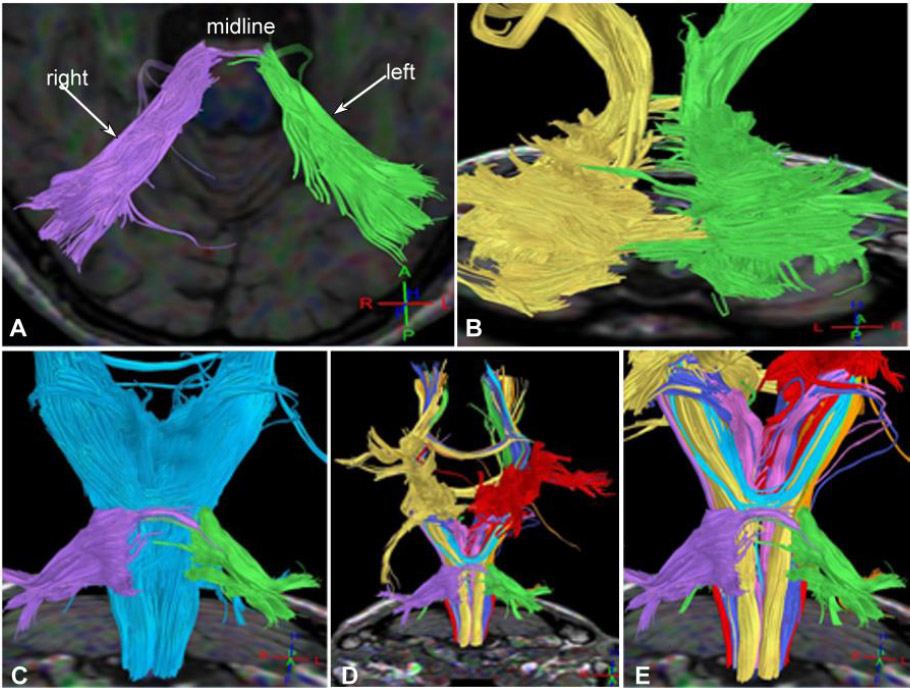
Interrupted and destroyed cerebellar peduncle, damaged arcuate fibers, interrupted cerebellar peduncle, and projective fibers in case 4. (**A**). Bottom view: The cerebellar peduncle (purple on the right and green on the left) was interrupted and destroyed at the midline of the pons. (**B**). Back view: The arcuate fibers (yellow on the right and green on the left) were severely damaged in both cerebellar hemispheres, which was correlated with cerebellar atrophy. (**C**): Anterior view: The cerebellar peduncle (purple on the right and green on the left) was interrupted at the pons level of the projection fibers (blue). (**D, E**). Anterior view: The adjacent relationship was shown between the projective fibers and the cerebellar peduncle (D), and in the magnified view (E), the damaged cerebellar peduncle (purple on the right and green on the left) was demonstrated in the brainstem.

Significant interruption and destruction (Figs. **4**-**7**) were revealed in the midline of the cerebellar peduncle (Figs. **4A**, **5A**, **6A**, and **7A**), arcuate fibers in the cerebellum (Figs. **4B**, **5B**, and **6B**), and the spinothalamic tract within the brainstem and spinocerebellar tract (Figs. **4C** and **D**, **5C**-**E**, **6C** and **D**, and **7B**-**E**). Significant (*p* <0.05) decreases were detected in the FA values of the cerebellar peduncle (0.51±0.04 *vs.* 0.68±0.02), cerebellar arcuate fibers (0.37±0.08 *vs.* 0.51±0.05), spinothalamic tract (0.42±0.03 *vs.* 0.49±0.05) within the brainstem, and spinocerebellar tract (0.44±0.06 *vs.* 0.52±0.06) compared with those of the healthy controls [2]. Compared with the healthy controls, significant (*p* <0.05) increases were detected in the ADC values of the cerebellar peduncle (0.84±0.11 *vs.* 0.67±0.03), cerebellar arcuate fibers (0.87±0.12 *vs.* 0.66±0.05), spinothalamic tract (0.89±0.13 *vs.* 0.70±0.03) within the brainstem, and spinocerebellar tract (0.79±0.07 *vs.* 0.69±0.06) [2].

**Fig. (8):**
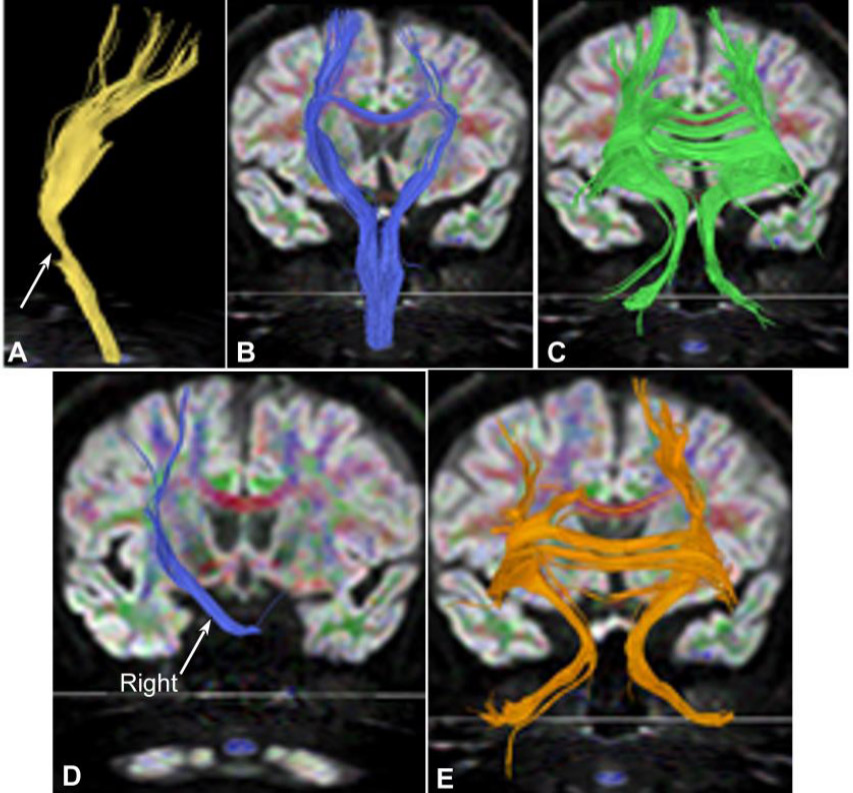
Left corticospinal tract interruption, lateral bundle of the corpus callosum, bilateral cortical dentate nucleus tracts, bridging fibers, and cortical cerebellar tract in case 3. (**A**). Left side view: The left corticospinal tract (yellow) was involved, with partial interruption (arrow) of the corticospinal tract at the pons level. (**B, C**). Back view: The lateral bundle of the corpus callosum (B, blue) and bilateral cortical dentate nucleus tracts (C, green) were mostly normal. (**D**). Back view: The bridging fibers (the right side is in blue, and the left side was damaged and was not shown) were interrupted on the left side of the brainstem. (**E**). Back view: the cortical cerebellar tract (orange) was normal.

**Fig. (9):**
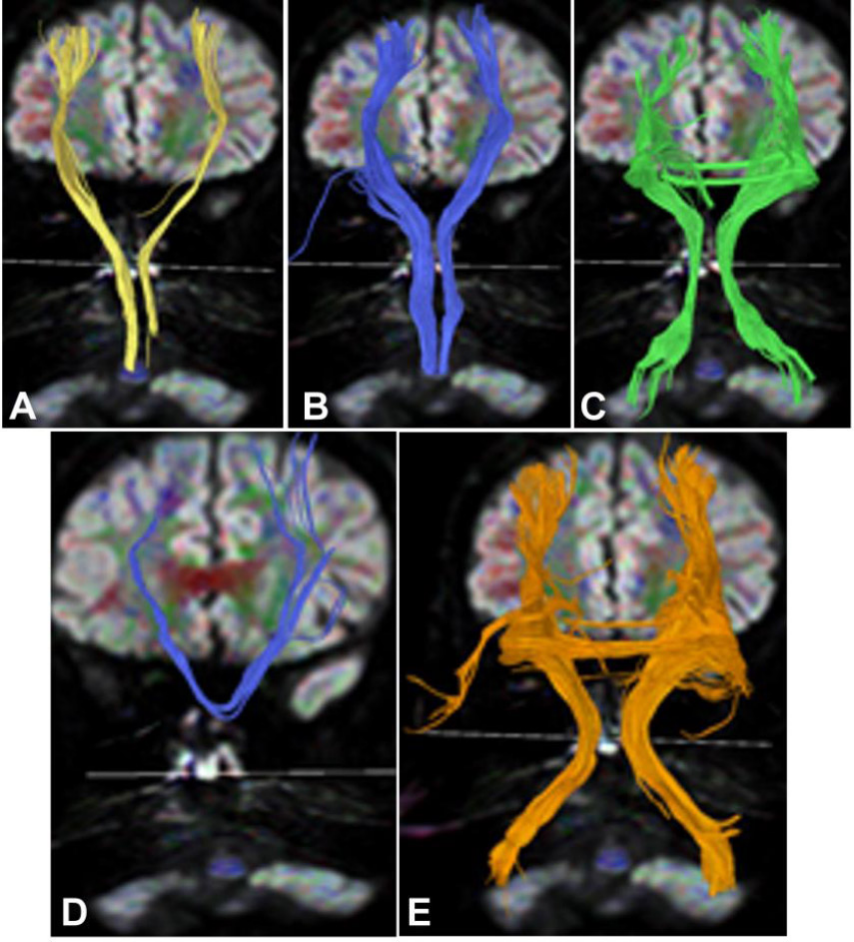
The left corticospinal tract in the brainstem, the lateral bundle of the corpus callosum, the bilateral cortical dentate nucleus tracts, and the cortical cerebellar tract in case 1. (**A**-**E**). Back view. The left corticospinal tract in the brainstem was interrupted (A, yellow on the left). The lateral bundle of the corpus callosum (B, blue), bilateral cortical dentate nucleus tracts (C, green), bridging fibers (D, blue), and cortical cerebellar tract (E, orange) were basically normal.

**Fig. (10):**
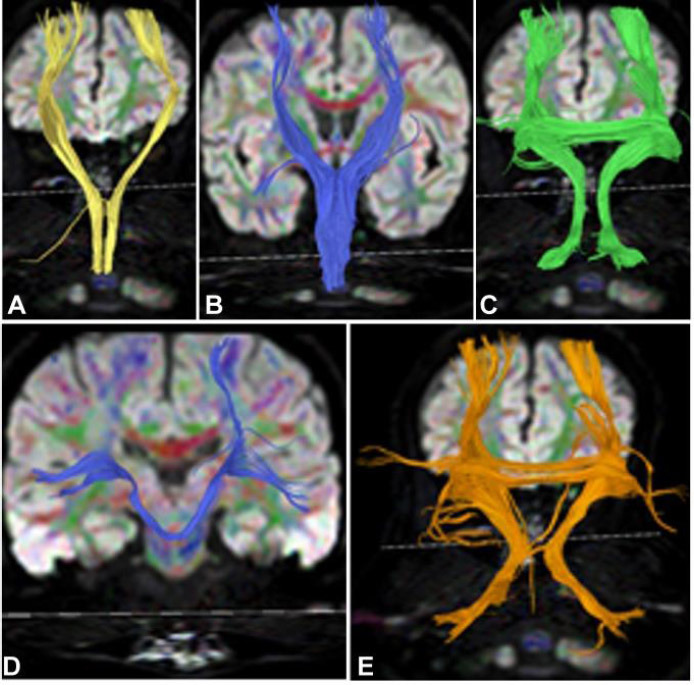
Normal fiber bundles in case 2. (**A**-**E**). Back view. The corticospinal tract (A, yellow), lateral bundle of the corpus callosum (B, blue), bilateral cortical dentate nucleus tracts (C, green), bridging fibers (D, blue), and cortical cerebellar tract (E, orange) were basically normal.

The corticospinal tract at the pons level (Fig. **8A**) and in the brainstem (Fig. **9A**) was interrupted, the lateral bundle of the corpus callosum and bilateral cortical dentate nucleus tracts (Fig. **8B** and **C**) were mostly normal, and the bridging fibers on the right side of the brainstem (Fig. **8D**) were interrupted. The cortical cerebellar tract (Fig. **8E**) was normal. The lateral bundle of the corpus callosum, bilateral cortical dentate nucleus tracts, and cortical cerebellar tract were generally normal (Figs. **8B** and **C**, **9B**–**E**, **10A**-**E**).

## 4 DISCUSSION

After investigating nerve fiber bundle damage in spinocerebellar degeneration using the DTI technique, it was found that the condition primarily involves the arcuate fibers and cerebellar peduncle within the cerebellar nerve fiber bundles, with partial involvement of other cerebellar fiber bundles. MRI DTI can clearly demonstrate both the extent of cerebellar fiber bundle damage and the spatial relationships between the fiber bundles entering and exiting the cerebellum in patients with spinocerebellar degeneration, thereby providing valuable information for further clinical research on the etiology and diagnosis of these disorders.

Hereditary spinocerebellar ataxia is an autosomal dominant neurodegenerative disorder with onset typically in middle age, characterized by progressive ataxia. It primarily affects the spinal cord, cerebellum, pons, and inferior olivary nucleus and is marked by neuronal atrophy and degeneration, loss of the myelin sheath, and mild glial proliferation, leading to widespread degeneration of the cerebellar hemispheres, vermis, and lower cerebellar peduncle, as well as the loss of Purkinje cells. The disease is most often caused by CAG trinucleotide repeat expansions encoding glutamine, with over 20 identified genetic subtypes. If one parent carries the mutation, each child has a 50% risk of inheriting the disease, regardless of sex. However, age of onset and severity may vary, even within the same family. While the precise cause remains unclear, most cases show a strong familial genetic predisposition. Patients with onset before 20 years of age are usually autosomal recessive, whereas those with onset after 20 years are predominantly autosomal dominant, with symptoms that progressively worsen. Genetic testing is the primary diagnostic method.

Clinically, the disease progresses through stages. In the early stage, patients develop unsteady gait, limb tremors, delayed responses, and reduced coordination. In the middle stage, they often exhibit slurred speech, impaired voice control, diplopia due to irregular eye movements, increased muscle incoordination, and difficulty with writing. Dysphagia and coughing during meals are also common. In the late stage, speech becomes extremely unclear or absent, with profound limb weakness and inability to stand, often requiring wheelchair use. Cognitive decline ensues, with eventual loss of consciousness and progression to a near-vegetative state.

In SCA12 patients, computed tomography and brain MRI have demonstrated atrophy of both the cerebellum and cerebral cortex, characterized by generalized cortical atrophy with moderate ventriculomegaly. At the same time, the basal ganglia and brainstem structures remain largely preserved [9, 13]. Neuropathological findings from the SCA12 proband revealed cerebellar and cortical atrophy, accompanied by mild pontine involvement, with moderate to severe loss of Purkinje cells and degeneration of the cerebellar granule cell layer [9, 13]. Neuronal intranuclear inclusions were identified in dopamine neurons of the substantia nigra, Purkinje cells, and, less frequently, in motor cortical neurons. Diffusion tensor imaging further showed that in SCA12 patients, the apparent diffusion coefficient (ADC) was significantly elevated, whereas fractional anisotropy (FA) was markedly reduced [9].

In patients with SCA1 and SCA2, MRI has qualitatively demonstrated atrophy and signal intensity abnormalities in the brainstem and cerebellum, as well as supratentorial involvement [9-12]. Neuroimaging studies have consistently reported grey matter atrophy in the hindbrain, cerebellum, insula, thalamus, parahippocampal gyrus, precuneus, and fronto-temporo-parietal cortices in SCA2 [14, 15]. White matter alterations in SCA2 have been shown to involve the cerebellum, thalamus, brainstem, cerebellar peduncle, internal capsule, corpus callosum, and frontal cortex [16-18]. On DTI, SCA2 exhibits significant degeneration in the bilateral anterior thalamic radiation, corticospinal tract, inferior fronto-occipital fasciculus, superior and inferior longitudinal fasciculi, uncinate fasciculus, cingulum, corpus callosum, and forceps major and minor, along with demyelination of the superior cerebellar peduncle and cerebellar white matter [18]. Pathological investigations of SCA2 have revealed a pattern of neuronal loss, myelin loss, and gliosis [19, 20]. On DTI, the ADC was significantly increased in the middle cerebellar peduncle and hemispheric white matter in SCA1, and in all examined regions in SCA2 [2]. Moreover, the ADC was consistently higher in SCA2 than in SCA1 across all regions. The FA was significantly decreased in both SCA1 and SCA2, but the reduction was more pronounced in SCA2, particularly in the transverse pontine fibers and corticospinal tract at the cerebral peduncle level. Although DTI did not allow for reliable differentiation between SCA1 and SCA2, significant differences in ADC and FA values were detected when compared with the control group [2].

In SCA3, Morphometric MRI demonstrated a typical pattern of atrophy or volume loss in the brainstem and cerebellum, with extensive lesions in some supratentorial areas. In contrast, DTI detected widespread microstructural changes in the brain's white matter, indicating disrupted brain anatomical connectivity [21]. In SCA 7, neuropathy is characterized by axonal loss in the white matter of the brainstem and cerebellum. Increases in the ADC and decreases in the FA values in the brainstem and cerebellum in SCA 7 suggest degeneration of the white matter in these areas [22]. In SCA 6, significant decreases have been observed in the volumes of the decussation of the superior cerebellar peduncles and inferior cerebellar peduncles, but significant increases in the ADC in the noncrossing superior cerebellar peduncles, middle cerebellar peduncle, and inferior cerebellar peduncles, which indicates degeneration of the cerebellar peduncles [23].

DTI is a functional MRI technique that utilizes the influence of the microenvironment on water molecules in human tissues. The diffusion motion of water molecules varies across different directions, with unequal distances of diffusion, and this motion is manifested as anisotropy. DTI is an advanced MRI method developed from diffusion-weighted imaging to observe the diffusion of water molecules. It applies sensitive gradients in at least 32 non-collinear directions, collects motion information of water molecules in three-dimensional space, quantitatively analyzes their diffusion characteristics, measures the microstructural properties of white matter fibers, and determines the spatial orientation of white matter fiber bundles. It is non-invasive, painless, and rapid, making it a powerful tool for studying the correlation between imaging and pathophysiology and for better understanding the relationship between brain structure and human behavior. This study, however, has several limitations, including its retrospective and single-center design, small patient sample, enrollment of only Chinese patients, and absence of follow-up, all of which may affect the generalizability of the findings. The retrospective design relied on pre-existing data and did not control for all potential confounding factors, which could have introduced bias. A single-center design also limits external validation, which is essential for confirming the study outcomes. In addition, the use of a 1.5T MRI scanner for DTI acquisition may have resulted in suboptimal image quality and spatial resolution. Future prospective, randomized, multi-center studies with larger and more ethnically diverse patient samples, and utilizing advanced MRI scanners with higher field strength, are needed to improve imaging quality, spatial resolution, and overall reliability of outcomes.

## CONCLUSION

To sum up, spinocerebellar degeneration is characterized by progressive degeneration of the cerebellum and its afferent and efferent pathways. As the cerebellum is a key structure of the central nervous system, its impairment commonly results in motor and balance disorders. Diffusion tensor imaging (DTI) allows a comprehensive assessment of the extent of fiber bundle damage within the cerebellum and the spatial relationships of fibers entering and exiting it, thereby providing valuable information for future clinical research on the etiology and diagnosis of these diseases.

## Data Availability

All data generated or analyzed during this study are included in this published article.

## LIST OF ABBREVIATIONS

MRI: Magnetic resonance imaging
DTI: Diffusion tensor imaging
SCA: Spinocerebellar ataxia
FA: Fractional anisotropy
ADC: Apparent diffusion coefficient

## STANDARDS OF REPORTING

STROBE guidelines were followed.
